# Intelligent Network Applications Monitoring and Diagnosis Employing Software Sensing and Machine Learning Solutions

**DOI:** 10.3390/s21155036

**Published:** 2021-07-25

**Authors:** Marius Minea, Cătălin Marian Dumitrescu, Viviana Laetitia Minea

**Affiliations:** 1 Department Telematics and Electronics for Transports, University “Politehnica” of Bucharest, 060042 Bucharest, Romania; 2 Department Technology, Orange Services Romania, 020334 Bucharest, Romania; viviana.minea@stud.etti.upb.ro

**Keywords:** software sensing, preventive failure maintenance, Apdex performance index, intelligent agent, wavelet decomposition, undecimate wavelet transform, Hurst exponent

## Abstract

The article presents a research in the field of complex sensing, detection, and recovery of communications networks applications and hardware, in case of failures, maloperations, or unauthorized intrusions. A case study, based on Davis AI engine operation versus human maintenance operation is performed on the efficiency of artificial intelligence agents in detecting faulty operation, in the context of growing complexity of communications networks, and the perspective of future development of internet of things, big data, smart cities, and connected vehicles. (*). In the second part of the article, a new solution is proposed for the detection of applications faults or unauthorized intrusions in traffic of communications networks. The first objective of the proposed method is to propose an approach for predicting time series. This approach is based on a multi-resolution decomposition of the signals employing the undecimate wavelet transform (UWT). The second approach for assessing traffic flow is based on the analysis of long-range dependence (LRD) (for this case, a long-term dependence). Estimating the degree of long-range dependence is performed by estimating the Hurst parameter of the analyzed time series. This is a relatively new statistical concept in communications traffic analysis and can be implemented using UWT. This property has important implications for network performance, design, and sizing. The presence of long-range dependency in network traffic is assumed to have a significant impact on network performance, and the occurrence of LRD can be the result of faults that occur during certain periods. The strategy chosen for this purpose is based on long-term dependence on traffic, and for the prediction of faults occurrence, a predictive control model (MPC) is proposed, combined with a neural network with radial function (RBF). It is demonstrated via simulations that, in the case of communications traffic, time location is the most important feature of the proposed algorithm.

## 1. Introduction

With the rapid growth of the communications networks and their large involvement in applications such as internet of things, big data, smart cities, and connected vehicles, the importance of these functional components becomes critical, and the need of their resilience also increases rapidly. Traditionally, faults in the communications networks are approached manually as a large part of network management activities, but the continuous increase in networks’ complexity consequently leads to more demanding and intensive network management activities; thus, the human component needs to be assisted or supported by automated processes of detection and fault management. Expert systems have been implemented in many applications, including fault management, but sometimes the complexity of applications prevents an effective use of these solutions. Moreover, in a predictive maintenance policy, there is also an increased need of automated processes to detect applications overloads, which does not represent a fault but may trigger malfunctions of different components of a network or specific service, or lower response times. In large geographically deployed systems including communications networks, such as smart cities, or connected vehicles, several services are critical, involving zero tolerance in delayed messaging. According to these reasons, we believe that a flexible solution for application monitoring in a complex network, by using software detection and being capable of learning behavior of the different components may address the specific problems of resilience required by the preventive maintenance. Automation of such activities shall be assisted by machine learning or other artificial intelligence technologies. The benefit will be the ability to produce records of faults and solutions evolution over time, helping designers to enhance the network structure, modules, and applications. Below are several envisaged tasks regarding new solutions performed:

- ability to operate with large amounts of new and dynamic data;
- ability to enhance performance by learning from experience;
- ability to accept scalability with respect to information types, domains, and structures;
- ability to be friendly with modularity, both in hardware and software, requiring minimal maintenance activities when the domain knowledge changes;
- ability to use new information when performing correlation between faults and remote application monitoring;
- ability to produce intelligent alerting for the on-call administrator.

Considering the above-mentioned directions, the objectives of this work include the following:

- to assess the basic elements that are to be taken into consideration when developing a more complex structure for the fault maintenance system (FMS), based on a hierarchical structure of intelligent agents;
- to analyze a typical case study, taken from a real mobile communications network operation, in order to estimate the efficiency of AI engines in a constantly monitoring level of service of applications and abnormal behavior;
- to propose a new approach in developing and structuring the AI engines (or agents) for the FMS of a communications network in order to cope with continuous growth of complexity and variety of functional components and applications, easing the work of human operators.

Our proposal for the detection of applications faults (or unauthorized intrusions) in the traffic of communications networks is based on the analysis of traffic long-range dependence. The estimation of the long-term degree of dependence is performed by estimating the Hurst parameter of the time series analyzed by decomposing undecimate wavelet multiresolution.

Unlike the discrete wavelet transform (DWT), which reduces the sampling of approximation coefficients and detail coefficients at each decomposition level, the undecimate wavelet transform does not incorporate downward sampling operations. Thus, the approximation coefficients and the detail coefficients at each level have the same length as the original signal. UWT also exemplifies the coefficients of low-pass and high-pass filters at each level. The sampling operation is equivalent to the expansion of the waves. The resolution of the UWT coefficients decreases with increasing level of decomposition. This association between the UWT and the Hurst parameter is a relatively new statistical concept in communications traffic analysis. To achieve the algorithm, we started from the following hypothesis: an observed time series is generally considered to be decomposed into a signal, corresponding to the state of a process that describes the system of interest and noise. For time series dominated by stochastic properties, the Hurst parameter is a simple means to characterize the dependence of observations separated in time, and as a reference we associate the signal corresponding to the traffic of a communications network as white noise. The presence of LRD in network traffic has a significant impact on network performance, and the occurrence of LRD may be the result of anomalies that occur during certain periods. To control the proper functioning of a communications network, a predictive control model is proposed, based on a neural network of a radial function type (MPC-RBF), which employs the results obtained by decomposing an undecimated wavelet and calculating the Hurst parameter. For training the RBF network, the random input-output signals of the MPC are used, because an important problem in building the neural network is to obtain an optimal training, which must improve the generalization and reduce the necessary number of training samples. The MPC-RBF model is used to estimate the future behavior of the communications network. Table 1 presents applications of the wavelet transform for the analysis of traffic communications.

As shown in Table 1, there are no references in the literature on the use of the undecimated wavelet transform in the analysis of network communications traffic. The correlation of the UWT decomposition with Hurst parameter also represent a novelty in this field.

It has been demonstrated via simulations that, in the case of communications traffic, time location is the most important feature of the proposed algorithm.

All the continuously evolving requirements of the communications networks induce the idea that a human-operated maintenance service tends to become more difficult, therefore there is a need of involvement of an automated, artificial intelligence-based process. The remaining part of this paper is structured as follows: Next, Section 2 is dedicated to a study on similar state-of-art work, Section 3 performs an evaluation on the existing instruments for online applications and faults management, Section 4 is dedicated to a case study on the efficiency of FMS automation in a mobile communications network, Section 5 is a proposed solution, and finally, conclusions.

## 2. Related Work

Studies in the domain of preventive maintenance came into actuality when the complexity of communications networks increased significantly, and the transition to digital, static switching replaced the traditional, relay-dependency public switched telephone networks. Presently, with the growing pressure put by the rapid deployment of IoT, 5G, and big data technologies, an increased number of applications need specific requirements for communications, including connected vehicles, smart cities, green energy applications, e-services, etc. There are many studies in this area, and numerous solutions have been proposed, developed, and tested. However, the research is still open in this direction and there are difficult requirements, sometimes contradictory, that must be satisfied. In dense urban areas, many devices need permanent, high-responsive wireless connection to the internet as there are many sensors. Access points and the communications channels are in high demand, in terms of link reliability, speed, latency and bandwidth. The communications infrastructure is required to be highly available, with similar requirements for robustness, and resilience. The research in this direction is promising and growing, having the purpose of increasing overall experiences in network fault management. The authors of [8] propose a dedicated flow of fault management consisting of alarm detection, customer satisfaction data collection (regarding the immediate action on the alarm), alarm filtering and correlation (classification into physical or software), fault diagnosis via analysis and testing, correction plan elaboration, alarm recovery verification, and recording features (timestamp for detection, recovery, or other). They propose the classification of alarms into two groups, physical and logical. They offer solutions such as neural networks or bayesian belief networks filtering and other artificial intelligence-based solutions to handle the detection of faults. A condition monitoring subsystem combined with structural health solutions succeeded by averaging techniques and the Daubechies wavelet for eliminating HF disturbances are evaluated in [9] for preventive maintenance, followed by denoising and compression based on discrete wavelet transform (DWT). The process of locating a network fault is a difficult task and several solutions are also shown in this direction in [10]: definition and storage of fault isolation specific rules, layered stack of rules, network configuration database for reconfiguring alternate routes with level of service monitoring, followed by decisions regarding the healing methods. The same attention is given in [11] on the faults of a system, signals’ analysis, and artificial neural networks (ANNs) based methods by employing transient data extracted from the fault voltage and current waveforms for recovering a faulty energy supplying system. Chang C-W et Co. [12] consider a study on the artificial intelligence algorithms used in smart machine tools’ fault management classifying and presenting conclusions of over 160 scientific works in this domain. An interesting solution related to a centralized fault management system for 6LoWPAN WSN (IPv6 over low-power wireless personal area network) is presented in [13], based on a two fault detection levels approach: a local level, based on sensors mounted in the network and employing statistical methods, and a second level, processed by the base station with the help of multi-layer perceptron-type artificial neural networks (ANN) classifiers. These are presented in a comparative manner, both centralized and decentralized approaches for fault management. Fault management of cellular communications networks is considered by R. Shaffin et al. [14], the authors present their opinion on the difficulty of the introduction of artificial intelligence in 5G and next generation networks, with the purpose of solving major technical barriers in terms of performance, robustness, and growing complexity. They propose the use of specific KPIs (key performance indicators) consisting of performance management (PM) counters sent periodically, for monitoring-specific processes. In the field of image processing, which becomes more attractive for different domains, work [15] proposes a DWC—mixed hardware and software replica (duplication) with comparison and triple modular redundancy (TMR) to obtain higher degrees of fault detection and fault tolerance. [16] describes the usage of ANNs for early detection of failures in power systems. Two types of ANNs are employed: back propagation (BP) and genetic algorithm (GA) for early fault detection (EFD). An interesting work has been found in [17], in which T. Chalermarrewong, S. See, and T. Achalakul propose a combination of two concepts, prediction and migration, as a more efficient solution for emphasizing the system’s degree of availability, based on a prediction of a node failure and possibility to migrate its job to another node, applicable for large networks of computers. The authors present the concept of ARMA, a prediction with an autoregressive-moving-average-model. The paper focuses on the possibility to predict a failure of a datacenter with monitoring on hardware faults. For other industrial applications, such as electrical machinery, [18] evaluates different applications of expert systems (ES), artificial neural networks (ANN), fuzzy logic systems (FLS), and genetic algorithm (CA) techniques. The authors write that these systems can be integrated together and with other similar techniques. They focus mainly on four of these diagnostic tools, artificial neural networks, knowledge-based systems, fuzzy logic systems, and genetic algorithms designed to work with fault management systems of electric machinery. In [19], the authors consider the importance of active fault management in autonomous systems. The proposed solution tries to optimize the fault diagnosis procedures employing sensitivity analysis. It has proven its efficiency in both fault excitation and fault mitigation in an autonomous system, such as the electrical power train of an autonomous vehicle. In the same area of interest, connected vehicles represent the future of automotive, and work [20] proposes a fault-tolerant cooperative motion planning method for a cloud of connected vehicles designed to a more flexible driving, thereby promoting the throughput to reducing congestions. The solution relies on a parallel computation framework, which enables to keep solving the original goals as much as possible when faults have partially degraded the vehicle cloud’s work capability. A preliminary fault tolerant distributed state estimation scheme for complex networks is developed by F. Tedesco, G. Franze, and A. Cassavola in [21]. A distributed sensors architecture is proposed for network-based systems with distinct groups of nodes, such as plants, sensors, and computational agents. The solution is based on the trust that agents form on the quality of the measurements to select the most appropriate sensors for state estimation. A comparison between different types of wireless communication technologies suitable for IoT and smart cities is presented in [22], with emphasis on advantages and disadvantages, including lifespan analysis and reliability. In work [23] a failure processing algorithm of a power backbone communications network is proposed, based on state perception and artificial intelligence. The authors deliver a fault recovery algorithm based on reinforcement learning for a power backbone communications network service. Cyntia S. Hood and C. Ji [24] focus on the proactive fault detection processes and conclude that it is possible to use adaptive statistical methods to detect network faults without using models of specific faults. In the same direction, [25] describes a dedicated, intelligent, and general fault management service designated for heterogeneous networks. Here, a centralized fault management system is proposed for the administration of heterogeneous networks. A tutorial and a best practice study on solutions for fault and performance management is described in [26] in the domain of virtual network services running on multiple clouds. Based on a study of over 80 reference papers, the work introduces the issue of managing the availability and performance of carrier services using NFV (network function virtualization) spread over a multi-cloud architecture. Network faults are diagnosticated via data mining procedures in [27], where a method of automated fault diagnosis is described based on decision trees, rules, and bayesian classifiers for visualization of network faults. The faults management in complex transport interacting systems that compose the underground metro infrastructure, based on machine learning, is evaluated in [28] and in [29], in which the authors present a distributed intelligent fault management (DIFM) system for communications networks. The solution is based on a distributed cooperative multi-agent system, with probabilistic networks as the framework for knowledge representation and evidence inferencing. A solution for supporting intelligent fault management, and performance operations for communications networks is described in [30]. Fault management automation via intelligent mobile agents is analyzed in the paperwork of [31,32,33], and deep Q-learning for self-organizing networks’ fault management and radio performance improvement is considered in [34]. Similarly, an online failure diagnosis for cellular networks, based on contextualized indicators is proposed in [35]. Authors of [36] created a survey based on over 100 reference papers on fault localization techniques for networks of computers and concluded that fault localization has a high degree of difficulty, resulting from complexity, unreliability, and non-determinism of communication systems. Fault localization in complex communication systems remains an open research problem. Similar analysis and solutions are presented in papers [37] to [36], with focus on cellular networks and wireless sensor networks. The difficult problem of discovering rules for fault management is addressed by R. Sterritt in [38]. Several authors propose a layered scheme for the fault management in complex networks, such as those for IoT [39] and a test scheduling with risk-sensitive criteria for triggering alarms [40]. Finally, for software-defined networking of the fault management is overviewed in [41,42] and general fault management techniques employing artificial intelligence are considered in [43,44]. As it can be observed, there is a wide palette of research and direction in this area, and the field is still open to new approaches, due to its vastity and complexity.

Table 2 summarizes the methods used in the literature for anomaly detection.

## 3. Existing Instruments for Online Applications and Faults Management

### 3.1. Overview

Management of large networks comprising many elements comes with difficulty, growing with the extension and diversification of the network. There are both hardware and software functional components that must be periodically checked for health and workload. In a preventive maintenance scenario, algorithms and analyses are to be performed with the use of key performance indicators, such as level of service, customer satisfaction etc. for software components, or monitoring for temperatures, fan RPM counting, energy consumption or other health indicators for hardware functional components. As the structure of the network complicates, due to redundant components (needed to increase reliability), the procedures also become increasingly sophisticated, rendering manual administration a challenging task, potentially impossible, without the benefits of automation. Several directions of research are opened in the field of artificial intelligence and machine learning involvement in this direction. Presently, there are several industrial applications available for economic usage. In the following, a brief survey on these applications and their advantages and drawbacks:

- WaitListCheck is an effective solution for online application management dedicated to public housing authorities, that allows:
  ○ online checking of duplicate applications;
  ○ direct communication with applicants;
  ○ timestamping of received information.

- SALESmanago CDP with AI—an application for the management of commercial activities involving teams:
  ○ contacts management (CRM);
  ○ command center;
  ○ web push notifications;
  ○ anonymous marketing automation;
  ○ machine learning and artificial intelligence marketing;
  ○ additional advanced marketing automation extensions etc.

- Dynatrace—represents a software platform based on components of artificial intelligence, designed to supervise, and optimize applications’ performances and development, the infrastructure for information technology, and to monitor and record users experience for large companies and service providers:
  ○ infrastructure monitoring (hybrid cloud observability, fault domain isolation, serverless, container, pod, and network);
  ○ applications and microservices (hybrid cloud distributed tracing, automatic code-level root-cause and profiling, front and back-end availability and performance);
  ○ applications security (run-time vulnerability detection, impact analysis, DevSecOps automation);
  ○ business analytics (real-time business insights, impact and conversion, BizDevOps integration and automation;
  ○ cloud automation (ecosystem integration, API programmability);
  ○ Davis AI—automatic mapping of digital ecosystem to create a topology. Collection of information and automatic error recognition and analysis, online monitoring of data transactions, detection of fault causes and contextual analysis, quantification of business impact.

Such types of applications are meant to ease the maintenance work of a complex network of mixed hardware and software modules but require an initial training in which complexity is directly proportional with the complexity of the system and applications.

### 3.2. Instruments to Determine Efficiency, Availability, and Level of Service (LoS)

The preventive maintenance is a difficult task to be performed by manual operation, mostly for extensive networks and systems with mixed hardware and software. If there is a public service, the user satisfaction should be considered regarding the response time of the requested service. This feature importance increases when critical aspects in messaging or internet data delivery processes are involved. However, presently several of these assessments may beneficiate from different types of instruments developed.

From the hardware point of view, in a remote-controlled system, different functional components may be managed via internet or other communication means, and their functionality can be checked via dedicated sensors, dedicated software sequences or simply by answer and response. The service availability in such an extended system is always a concern of the responsible owning authority, and an agreed measure for it is often a key performance indicator (KPI), as part of the IT service management. From the hardware point of view, a component’s availability for a definite period is determined by:

(1) $$A=\frac{\tau_{u}}{T_{m}}\cdot 100,$$

(2) $$T_{m}=\tau_{u}+\tau_{d}$$

where A represents availability in percents, $\tau_{u}$ is the period when the hardware component functioned at full parameters, $\tau_{d}$ is the period when the component was functioning in degraded state or not at all, and $T_{m}$—interval of interest.

From a service point of view, there is a slight difference in calculation:

(3) $$A=\frac{T_{m}-\tau_{d}}{T_{m}}\cdot 100$$

where $T_{m}$ represents an agreed service time. However, a service cannot be categorized as “not available” if it can still be accessed by someone. It must also be taken into consideration the satisfaction degree that the user feels when accessing a service in terms of delay, in which the service is able to respond. From the literature, a tolerated formula for this quantification is:

(4) $$A=\frac{\tau_{p}-\tau_{o}}{\tau_{p}}\cdot 100$$

where $\tau_{p}$ stands for potential interval of time with user satisfaction and $\tau_{o}$ is total time with user outages.

A more raffinate instrument in defining mixed hardware and service availability is the Application Performance Index (or Apdex (The Apdex Alliance is a group of companies that collaborated in establishing the Apdex standard)), which is expressed by:

(5) $$I_{A}=\frac{n_{s}+0.5\cdot n_{t}+0\cdot n_{u}}{N},$$

(6) $$N=n_{s}+n_{t}+n_{u}.$$

In the above equations, $I_{A}$ represents the Application Performance Index, which ranges from 0 to 1, $n_{s}$ is the number of satisfactory service level counts, $n_{t}$ the number of tolerable service level counts, and $n_{u}$ the number of unsatisfactory service level counts. N is the total number of samples.

From the applications availability point of view, for determining the impact it has on the other functional components, we propose a derived formula:

(7) $$NS=\frac{\sum_{i=1}^{m}\tau_{ci}+\alpha \sum_{j=1}^{n}\tau_{nj}}{T}$$

(8) $$T=\overset{m}{\underset{i=1}{\sum }}\tau_{ci}+\overset{n}{\underset{j=1}{\sum }}\tau_{nj}+\tau_{c}$$

where NS represents the Index of Non-Satisfactory Performance, m the total number of failures that produced total loss of service, $\tau_{ci}$ the duration of *i*th total failure of the service, n the total number of failures that produced partial loss of service features, $\tau_{nj}$ the duration of the jth order failure that produced partial loss of performance, $\tau_{c}$ total time when services were 100% available, α a weighting factor, and T the total interval of measure. The weighting coefficient α is chosen according to the importance of the functional component or sub-service that has not been functional for the given period of interest.

**Figure 1:**
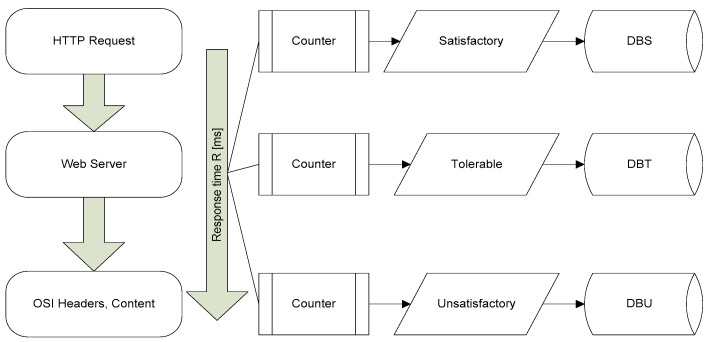
Process for determining Apdex Index. DBS: database with number of satisfactory response times; DBT: database with number of response times within tolerable limits; DBU: database with number of unsatisfactory response times.

Considering the level of service (LoS), several criteria are defined for evaluating this important feature to preventive maintenance in communications [45]: availability, response time, capacity, capability indicators, support, and reversibility. With the communications networks involved in smart cities, connected vehicles, and other IoT applications, an important issue is security. For this sector, the most important KPIs are reliability, authentication, cryptography, security, logging, auditing, vulnerability, and service changing. Consequently, we consider that the development of an automated process for monitoring several, if not all these aspects is extremely important for the flawless operation of complex communications networks involved in IoT. In relationship with equation describing NS, the weighting coefficient α may be adjusted according to the service it refers to and its importance in the flow of network functionalities. In Figure 1 is shown the process for determining Apdex Index.

There are certain applications that make this evaluation process in an automated manner, presenting graphically the evolution of the application/component service level. While still widely employed, the Apdex represents a static methodology to determine the application or system’s level of service. With the extensive grow of complexity, however, it is expected that this solution may prove insufficient in the near future. Therefore, we propose to develop this methodology by adding a dynamic feature to it. When analyzing the reliability of a large system, such as a communications network providing different types of services, a statistical filtering of failures may help determining main causes provoking chain failures of different functional components [46,47,48,49]. An in-depth analysis of the triggering event that caused a chain failure of several elements finally inducing a serious level of service degradation should be useful in preventing such future events. However, determining correlations between those events may be difficult for manual processing in extended equipment structures. Therefore, making use of machine learning in this domain is recommended. The proposed solution comes in line with other, similar initiatives, presented in the first part of this work.

## 4. Case Study—Efficiency of FMS Automation for a Mobile Communications and Services Provider

### 4.1. Overview

**Figure 2:**
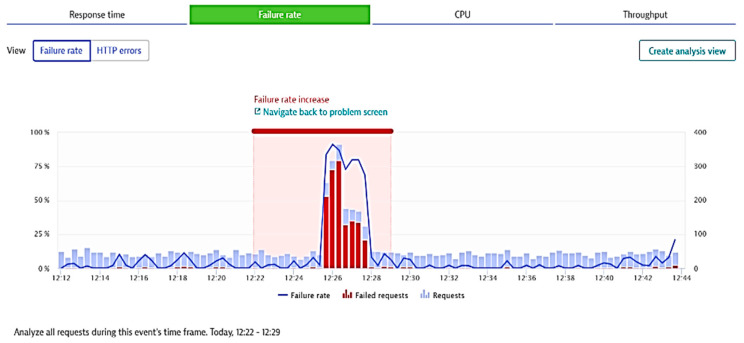
Caption of a failure rate increase detection as shown by Davis AI.

**Figure 3:**
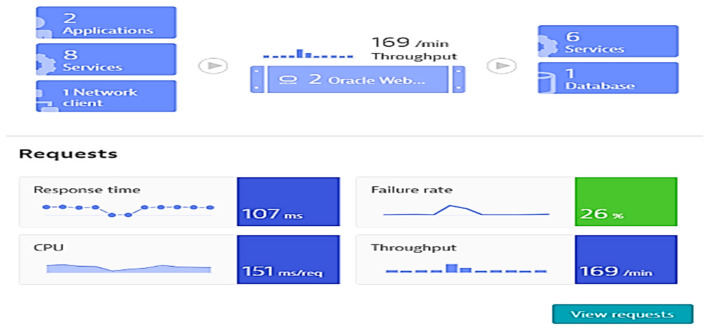
Monitored parameters and affected applications as shown by Davis AI for the specific case study.

For this case study, a mobile communications service provider was chosen to evaluate the efficiency of its FMS and perform some level of service tests. The service provider is equipped with a specific current Apdex value for each monitored application, a specific target for the Apdex value, and a specific availability threshold. The availability index is automatically established according to the time the respective application is up, compared to the measuring interval. Dynatrace Davis employs AI to monitor logs of applications (by specific keywords). The following is a list of the correspondent tests performed by Davis AI:

- analyzing a specific function up to code level:
  ○ Response time (ms),
  ○ Failure rate (%), as shown in Figure 2,
  ○ CPU response time (ms/req), as shown in Figure 3; req: request,
  ○ Throughput (req/min).
- performing database calls;
- outgoing requesting to other applications;
- monitoring the response times across requests during the selected period, including processors and memory loads;
- performing business impact analysis based on counted dependencies (e.g., impacted users by a failure, and affected service calls), displaying percentage of functions and applications requests affected;
- possibility of setting specific tests according to user preferences (by employing automated bots to perform tasks);
- suggesting failure root cause for the monitored applications, based on time correlations and analysis of all transactions that consider the impacted components.

**Figure 4:**
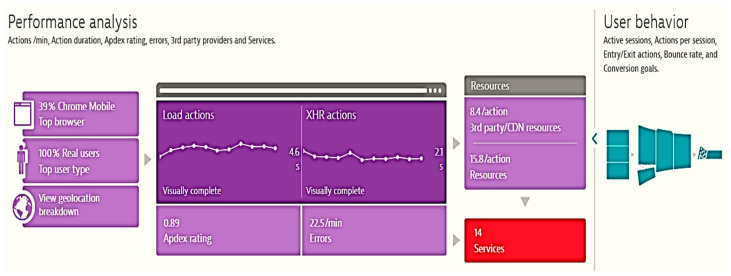
Extra information shown in application performance monitoring.

The instrument is also able to display performance analysis (Figure 4) based on user behavior, showing Apdex index, used browser type, errors encountered, and availability of resources [50,51,52].

**Figure 5:**
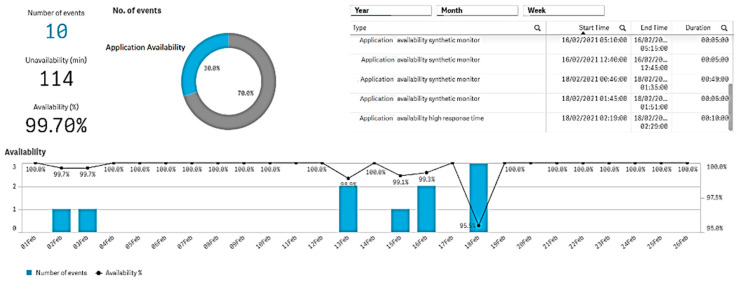
Displayed information regarding the availability of an application.

The availability index is displayed based on Davis AI data and a specific application is monitored in terms of number of events, unavailability time, availability percentage, and associated events shown in a synthetic monitoring window (Figure 5). Here it is shown that a specific application has encountered 10 events in the monitored period, totaling 114 min of unavailability. The availability percentage has variations between 100% and 95.5% (lowest level), with variable durations at the recorded moments of failures. The AI engine records start and end times of each failure (right side of the figure). The amplitude of failures is also shown (number of simultaneous events)—the vertical light blue bars. Thus, the advantage here is that the Davis AI engine monitors both time position and gravity of the fault (by the number of simultaneous events).

The processes’ recovery activity, in case of a major failure detection, consists now in manually checking all possible causes shown in Davis AI information that fed the application availability index. This represents a time-consuming process, involving human resources and several investigations to determine the real cause of the failure. Occasionally, the process can be shortened if the maintenance personnel is instructed with specific procedures, or there are specific alerts configured on the most known servers holding the applications. For example, in the case study, based on Davis AI measurements and operations for four months (from February to May), it resulted in a 99.94% availability (mean time between failures MTBF = 0.99941550) for a single specific application. However, in the administration of a complex communications network, there are over 1000 inter-related applications, and the overall MTBF may suffer from different causalities. Table 3 below shows availability percentages recorded for top-ten critical applications (as importance) in the case study by Davis AI, performed for a local communications operator.

For long-term network preventive maintenance purposes, or for improving its reliability and resilience, it will be useful to develop an over-imposed AI application to monitor all failures causes and perform correlations between causes and effects, in order to keep record of major connections between failures and their effects in network services operation. Another added value will also be for the understanding of causes that produced a low availability and Apdex indexes over longer periods of time (at least a week, for example). This may be an important argument for reporting activities and future improvement of network resilience.

### 4.2. Specific Failure Case Analysis

**Figure 6:**
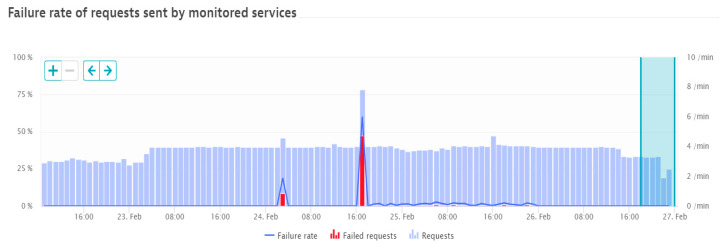
Failure rate of requests in a normal operating period.

**Figure 7:**
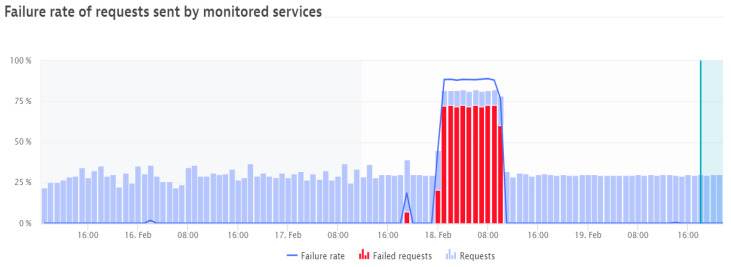
Failure rate of requests in a service breakdown period.

This case is analyzed for a specific communications’ services provider from Romania. The diagram in the figure below represents the number of requests per minute recorded for the specific application. The service is also monitored from the point of view of successful and failed requests. Figure 6 presents a period with usual service levels (within normal range). The application is sampled every minute and the number of requests (successful and unsuccessful, if any) are recorded per minute. The period for which the service levels in Figure 6 are analyzed is one week, with two cases of failure, which usually fit in the normal operation threshold. Figure 7 shows a similar period with a major failure, which after the analysis of the cause that produced it, it appeared that was caused by a planned upgrading of a hardware component. However, as it can be observed in Figure 7, manually recovering the failure was time-consuming. In a normal situation, the user-defined monitoring alerts would have been automatically triggered and consequently sending SMS notifications to alert the on-call administrator. The administrator would then immediately engage in solving the issue. However, in the specific case shown in this example, the monitoring alert was manually configured too high, considering an old customer behavior; the application monitor was continuously displaying false positive alarms and causing spam. Due to this specific setting, the alert was acknowledged and disabled from notifications, leaving an open possibility for an incident to occur and pass unnoticed. This is exactly what happened (as shown in Figure 7), in which for an entire night the incident was active, but unacknowledged by the application administrator, causing a much longer period for service unavailability.

For determining the loss of service level caused by the incident in this specific case, let us consider the following equation:

(9) $$LoS_{A}=\frac{\sum_{i}^{m}\varphi_{i}\cdot Ns_{i}-\sum_{j}^{n}\varphi_{j}\cdot Nu_{j}}{\sum_{k}^{t}\varphi_{k\;\cdot }\;N_{k}}$$

where: $LoS_{A}$ is the level of service for a specific application, $\varphi_{i}$ frequency of requests for the i sample (requests/min), $Ns_{i}$ the number of successful requests that had the frequency, $\varphi_{i}$, $\varphi_{j}$ represents the frequency of requests for unsuccessful sample j (requests/min), $Nu_{j}$ the number of unsuccessful requests that had the frequency, $\varphi_{j}$, $\varphi_{k}$ is the requests number per observation, and $N_{k}$ the sample number.

Considering the cases shown above in Figure 6 and Figure 7, the following levels of service were reached:

- normal operating case: LoSa = 0.957295;
- degraded operating case: LoSa = 0.462295.

The low value of LoS in the second case is due mainly to the high frequency of unsatisfactory requests over a longer period, caused by disregarding the above-mentioned alarm. Also, considering a quality factor of services defined by:

(10) $$Q_{a}=\frac{N_{s}}{N_{u}}\;,$$

we obtain:

- normal operating case: $Q_{a}$= 0.972973;
- degraded operating case: $Q_{a}$= 0.823529.

## 5. The Proposed Approach

### 5.1. Initial Considerations

To be protected from similar cases, a definition of a new Apdex index is proposed, taking into consideration the succession of applications that may consequently fail after a specific application detection of a malfunction has occurred.

We also propose employing an automated self-testing algorithm enabled to perform:

- correlations between failures and applications that have influenced each other by failures and determining chains of events. This is a process proposed to be performed by a dedicated, or over-imposed AI feature of the application monitoring. The AI should observe and learn rule for the evolution of two elements:
  ○ User requests frequency, for selected applications—this event has specific patterns during night, and other values during daytime. Detection of an unusual event should occur when this frequency exhibits certain dynamic threshold. This dynamic threshold shall be established by the AI via a machine learning process;
  ○ Threshold of successful user requests versus unsuccessful ones (rate of success);
  ○ Monitoring the number of retries of the same user (frequency of retries).
- at the occurrence of an application failure, tracking the succession of secondary applications decrease in LoS, if any, and recording;
- automatically issuing of alarms, based on LoS thresholds detection of known applications, and suggesting possible effects of an eventual failure of the respective application.

**Figure 8:**
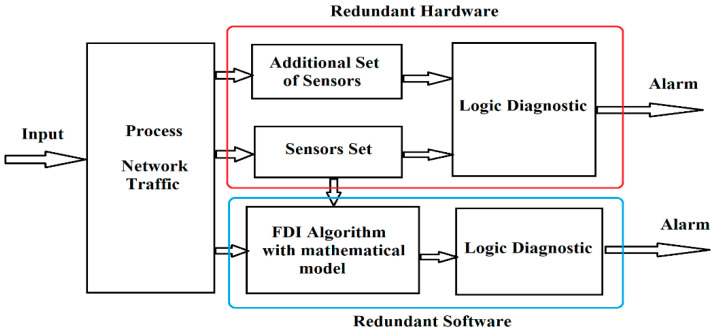
Hardware redundancy and software redundancy (analytical) applied for fault detection and isolation (FDI).

In general, diagnostic methods use the concept of redundancy, which can be of two types (Figure 8): hardware redundancy and software redundancy.

Hardware redundancy refers to the ability to compare duplicate signals, generated by various hardware sources, such as the result of measurements of the same signal obtained from two or more sensors. The techniques used in this approach are: signal processing methods (e.g., wavelet transform), limit testing (measurements compared to various thresholds indicating the presence of an anomaly), use of special sensors (limiting, designed to measure only certain parameters), and sensors placed in parallel for measuring the same parameter or using expert systems (based on “IF”- “THEN” rules for detecting an anomaly). Software (analytical) redundancy, proposed in this research, uses a mathematical model of the system along with other estimation techniques. In general, this approach does not require additional hardware resources and is usually more cost-effective compared to hardware redundancy. Conversely, analytical redundancy is more difficult to implement because it must ensure a certain degree of robustness in the presence of noise, perturbations or approximation errors introduced by the mathematical model. If these conditions are not considered, false alarms may be signaled in the presence of variations in input sizes or noise. By comparing the estimated values of the analytical model with those of the measurements obtained from the sensors, it is possible to detect and isolate the failures that appear in the process. The goal is to notice the difference between the model and the actual faulty system. The difference between the actual measured output of the process and the estimated output of the analytical model is called the residue. The residue value is compared with a threshold that can be either fixed or variable (e.g., adaptive threshold) after which it is determined whether a failure has occurred in the process.

### 5.2. Data Processing and Analysis

The goal of this research was to find a solution to the following requirement: Is it possible to determine the anomalies of functional components exhibiting abnormal operation in a data network and to estimate their health status, by analyzing the data traffic? This answer to that requirement is important for the design, operation, and failure maintenance of a network, and it can represent an explanation of why the network performance, measured in real time, is usually lower than the estimated performance in the design phase.

This concern has guided research toward the use of time series estimation algorithms. Estimating time series is currently a widespread method in research environments.

One of the major difficulties of the analysis of time series with long lengths (corresponding to a large volume of data) is the great complexity of the calculation. Complexity of calculations can be reduced by representation using multiresolution decomposition.

Such a representation may be obtained using the wavelet algorithm. The wavelet transform is used in the analysis of temporal events and involves a low computational complexity. In recent years, the wavelet transform has been used in many papers to analyze time series [53,54,55,56,57]. One of the main features of wavelet algorithms is the good determination of signals in time and space, especially for non-stationary signals, which have a high dynamic.

This method is based on transformed undecimated wavelet decomposition (UWT) and statistical time series analysis techniques by calculating the Hurst exponent. Based on the results obtained, a predictive control model (MPC) structure has been developed, which is based on a radial function neural network (RBFNN) used for modeling transfer functions and used for predicting the future behavior of the data network. The model proposed in this article is considered superior to the classic MPC variant because the RBFNN model is dynamically performing the real-time update for MPC.

#### 5.2.1. Analyzing Failure Detection with Undecimated Wavelet Transform (UWT)

The wavelet transforms of any signal $f\left(t\right)$ defined for any moment of time t≥0 is defined as [46]:

(11) $$WT_{f}\left(\sigma ,\tau \right)=\frac{1}{\sqrt{\left|\sigma \right|}}\overset{\infty }{\underset{-\infty }{\int }}f\left(t\right)\psi_{\left(\sigma ,\tau \right)}\left(t\right)dt$$

where $\psi_{\left(\sigma ,\tau \right)}\left(t\right)=\frac{1}{\sqrt{\left|\sigma \right|}}\psi \left(\frac{t-\tau }{\sigma }\right)$ with $C_{\psi }=\int \frac{\left|\psi \left(u\right)\right|}{\left|u\right|}du<+\infty$. The term σ represents a scaling factor, while τ is a translation factor. Common $\psi_{\left(\sigma ,\tau \right)}\left(\cdot \right)$ wavelet basis functions are the Haar wavelet, Symlets wavelet, Daubechies wavelet, and Mexican Hat wavelet [46]. The wavelet analysis algorithm uses threshold after the wavelet decomposition of the data into high and low frequencies to eliminate the high frequency signal. Anything above the threshold is eliminated. The final step of wavelet method denoising and multiresolution decomposition is the reconstruction of the signal. A discrete wavelet transform can be obtained by discretization of the scaling and translation factors.

Various studies have shown that a discrete wavelet transforms is an efficient mathematical method for analyzing network traffic flow signals experiencing transient and non-stationary phenomena for scale and resolution [58]. However, a decimated wavelet transforms down-samples the wavelet coefficients by discarding half of the data after each filtering stage. Furthermore, the decimated wavelet transforms coefficients do not have the shift insensitivity property down-sampling of signals may possibly lead to the loss of essential information. By employing the use of an undecimated wavelet transform, the shift insensitivity property of the coefficients is ensured, and the distortion induced by the down-sampler is eliminated [59].

The undecimated wavelet transform (UWT) W, using the filter bank of a 1-D signal $x_{0}$, leads to a set $W=\left\{d_{1},\ldots d_{J},c_{J}\right\}$ where $d_{j}$ are the wavelet coefficients at scale and j and $c_{J}$ are the scaling coefficient. The transition from one decomposition level to another is accomplished by [60]:

(12) $$c_{j+1}\left[n\right]=\left({\bar{h}}^{\left(j\right)}*c_{j}\right)\left[n\right]=\underset{k}{\sum }h\left[k\right]c_{j}\left[n+2^{j}k\right]$$

(13) $$d_{j+1}\left[n\right]=\left({\bar{g}}^{\left(j\right)}*c_{j}\right)\left[n\right]=\underset{k}{\sum }g\left[k\right]c_{j}\left[n+2^{j}k\right]$$

where $\left(h,g\right)$ represents the filter bank, * represents the convolution operation and $h^{\left(j\right)}\left[n\right]=h\left[n\right]$ if $n/2^{j}$ is an integer, and 0, otherwise. If we use a discrete filter in real-time it will have the impulse response $h\left[n\right]$, $\bar{h}\left[n\right]=h\left[-n\right],\;n\in \mathbb{Z}$ is its inverse version.

The undecimated wavelet transform is dyadic-orthonormal and provides the multiresolution analysis. Multiresolution decomposition is based on the multiscale approximation function.

At each scale level, the scaling function uses translation factors such as:

(14) $$\phi_{j,k}\left(t\right)=2^{-\frac{j}{2}}\phi \left(2^{-j}t-k\right)$$

The scaling function $\phi_{j,k}\left(t\right)$ is related to the scaling/approximation coefficients $c_{j,k}$, while the wavelet function $\psi_{j,k}\left(t\right)$ given by:

(15) $$\psi_{j,k}\left(t\right)=2^{-\frac{j}{2}}\psi \left(2^{-j}t-k\right)$$

is related to the wavelet/detailed coefficients. The approximation function is defined by the low-pass filters, and the wavelet function is defined by the high-pass filters.

The decomposition process of network flow traffic signals based on UWT-based MRA develops as follows: MRA analysis evaluates the signal concerning transient and non-stationary phenomena, decomposing it into sub-bands. For this research, we have tested several wavelet functions to determine the function that best approximates communications traffic for network anomaly detection.

**Figure 9:**
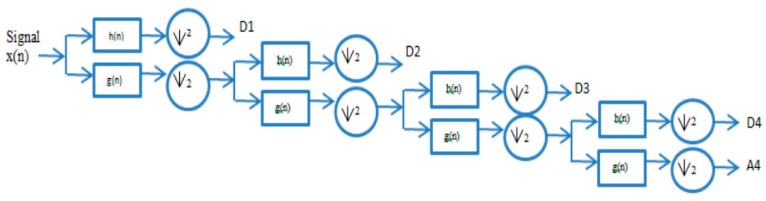
Decomposition multiresolution on four levels.

This procedure is presented in Figure 9. The method was implemented in the MATLAB software environment.

##### The Advantage of Using UWT

The resolution of the UWT coefficients decreases with increasing level of decomposition, for this reason a maximum of four levels of decomposition was chosen. For example, if we want to detect discontinuities by locating peaks in coefficients in a signal using level one decompositions using DWT and UWT, we find that all DWT detail coefficients on the first level are small, but by applying UWT, we find that the signal discontinuities on the first level are represented well due to the translation-invariant property. Another benefit of this transform is improved directional resolution. The near shift invariance and improved directional selectivity have facilitated excellent results in denoising, fusion, and other processing applications.

#### 5.2.2. Statistical Time Series Analysis Technique Employing the Hurst Exponent

The Hurst exponent is used in mathematics, especially in the analysis of chaos theory and in the spectral analysis of signals. Hurst exponent estimation, initially developed in hydrology, has been applied in fields ranging from biophysics to computer networks. However, the modern techniques used for estimation come from mathematics. The Hurst coefficient and the fractal size are in turn linked by the formula:

(16) D=2−H

The fractal size indicates the roughness of a surface. A low value Hurst exponent has a large fractal size and a rough surface while a small Hurst value has a small fractal size and a smoother surface. The Higuchi method calculates the fractal size of the samples Hurst exponent. To calculate the fractal size D, the Higuchi method requires a finite set of observations, using the interval *x*(1), *x*(2), …, *x*(N).

This consists in the formation of new waveforms by the interactive selection of different samples with the point starting m and delay factor k and a new one $x_{m}^{k}$ defined as follows:

(17) $$x_{m}^{k}=\left\{x\left(m\right),\;x\left(m+k\right),\ldots ,x\left(m+\left[\frac{N-m}{k}\right]k\right)\right\}$$

With m and k representing the reference time and the interval time used for the analysis, *m* = 1, 2, 3, …, *k*, and both k and m being integers. For a temporal interval equal to *k*, one gets k sets of a new time series. In our case, the surface is represented by the signal distribution, and a high value of the Hurst exponent indicates desynchronization. There are several methods that may be used to estimate the Hurst coefficient: the method of differential dispersion, the total dispersion method and the rescaled domain statistics method, the Higuchi method, the aggregate variance method, and the absolute moment method.

#### 5.2.3. The Method Proposed for Highlighting the Characteristics of Traffic Flow Employing Hurst Exponent and Multi-Resolution Wavelet Analysis

The proposed approach consists in combining the calculation methods of the Hurst exponent with the multi-resolution wave analysis. The algorithm follows these steps:

- a recording made during training is imported into the successive MATLAB program;
- the network traffic corresponding signals are extracted;
- the signals corresponding to the channels of interest are selected;
- the decomposition of the waves with multiple resolution is performed for the signals on each channel, successively using the Daubechies 2, Coiflet 4, and Symlet6 waves,
- for the signals decomposed into sub-components, the Hurst exponent is computed using Higuchi methods, with the 4th order detail coefficient and the 3rd order detail coefficient;
- the obtained values of the Hurst exponents are mediated on the number of test attempts;
- finally, the obtained values of the Hurst exponents are mediated on all records.

#### 5.2.4. Classification Using Radial Basis Function Neuronal Network (RBFNN) Based Model Predictive Control

For the specific classification in this research, the neural network with basic radial functions (RBF) predictive control-based model has been employed. Radial function-based neural networks (RBFs) have received increasing attention lately, due to their advantages. Compared to the MLP network, which tries to determine the minimum gradient of the error function, the process of the RBF network involves the approximation of an area in a multidimensional space that resembles that described by the input data. Considering this, the performance of neural networks based on radial functions is closely related to the ability to interpolate test data with data learned in the training stage. An artificial RBF neural network can respond better to a set of test data if it initially has multiple training vectors.

The RBF neural network has the following architecture:

- an input layer (sensory layer) composed of L virtual neurons (*i* = 0, …, L − 1), which does not perform a signal processing, but only a multiplexing, the actual processing taking place only in the intermediate layer, and output;
- an intermediate layer with M neurons (*j* = 0, …, M − 1), which implements the Gaussian activation function:

(18) zj=e−X−mj22σj2

- an output layer with N neurons (k = 0 … N − 1), which realizes the weighted sum of the outputs on the intermediate layer

(19) $$y_{k}=\sum_{j=0}^{M-1}w_{kj}z_{j}+\theta_{k}$$

where:

- *σ_j_* (dispersion) and m_j_ (average) describe centroid (prototypes of inputs);
- *X* = [x_0_, x_1_, …, x_i_, …, x_L−1_], the input vector;
- *x_i_* is the value assigned to the neuron i in the input layer, *i* = 0 … L − 1;
- *z_j_* is the exit of the neuron j from the hidden layer (intermediate), *j* = 0 … M − 1;
- *y_k_* is the output of the neuron k from the output layer, *k* = 0 … N − 1;
- *w_kj_* is the share of the connection between the neuron k in the output layer and the neuron j in the hidden layer (intermediate).

The partitioning of the input space into groups described by dispersion and average takes place in the hidden layer, while in the output layer the decision of belonging of the input vector to one of the classes is made. The number of neurons in the input layer is equal to the size of the input vector. The number of neurons in this layer may be less than or equal to the number of vectors in the training set. The optimal number of centroid (hidden layer neurons) is determined experimentally. The number of classes in which the classification is made sets the number of neurons in the output layer.

The vectors in the training set will designate the averages. It is desirable that the error on the drive lot is zero. Thus, a system of *MxN* equations with *MxN* unknown weights will be obtained, from which the weights will be determined.

The method of fixed centers (chosen at random) will be employed for the training of the RBF neural network. The positions of the centers of the functions are chosen randomly from the set of vectors in the training stage.

##### RBF Design for Our Proposal

A radial basis function arises naturally in problems of hyper-surface interpolation and approximation and in problems of learning input and output mappings from given sets of data. RBF networks usually have only one hidden layer, for which the combination function is based on the Euclidean distance between the input vector and the weight vector.

**Figure 10:**
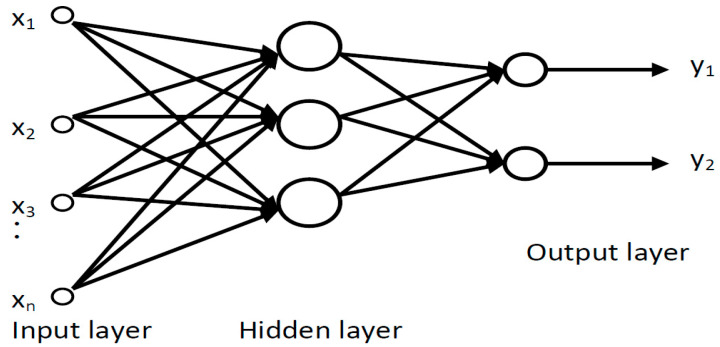
Neural network architecture used to classify network traffic flow signals (two-layer neuronal network).

The RBF network (Figure 10) having Gaussian function in the hidden layer has the ability to approximate any non-linear continuous function to an arbitrary degree of exactness.

The RBF neural network used in the methodology will have three layers (input, hidden, and output) and four nodes corresponding to the four levels of UWT decomposition.

#### 5.2.5. Design of the Predictive Control Model

The model-based predictive regulation methodology, known as MPC (model predictive control) has the following main characteristics:

(a) contains knowledge as accurate as possible of discretized regulated process (with sampling period T) dynamic model, which allows the estimation (prediction) of the response of the regulated process over a certain time horizon, called output prediction horizon. This is obtained by knowing the previous values adjusted, as well as past and future values (on the prediction horizon) of the control quantity and the disturbing quantity (if possible);

(b) allows development, on the time horizon of the output, of a “scale” control signal (constant over each sampling interval). This output signal is characterized by the sequence of N future commands. This ensures the optimal evolution of the process adjusted to the time horizon considered (e.g., obtaining a deviation as small as possible for the size adjusted to the reference conditions). At the same time, it is obtained a low power consumption, including for certain restrictions imposed on the control signal (input) and the regulation dimension (output);

(c) the effective implementation of only the first element of the calculated optimal sequence of commands, with the resumption of the whole process at the next sampling time (sliding horizon adjustment);

(d) the adoption of the “blocking” procedure, for blocking the hypothetical control on the last part of the prediction horizon, in order to simplify the predictive adjustment algorithm.

In the “blocking” procedure, the hypothetical command is kept free for the first M sampling periods from the N of the prediction horizon and is locked for the other N −M sampling periods at the last free value. Therefore, the number of distinct values of the command on the output prediction horizon is M. The interval [0, M] in which the command is free is called the free command horizon.

When building an MPC controller, the following information must be provided:

- prediction horizon (Np)—represents the totality of future samples based on which the MPC system predicts the output values;
- control horizon (Nc)—represents the totality of the prediction states based on which the MPC can influence the control.

**Figure 11:**
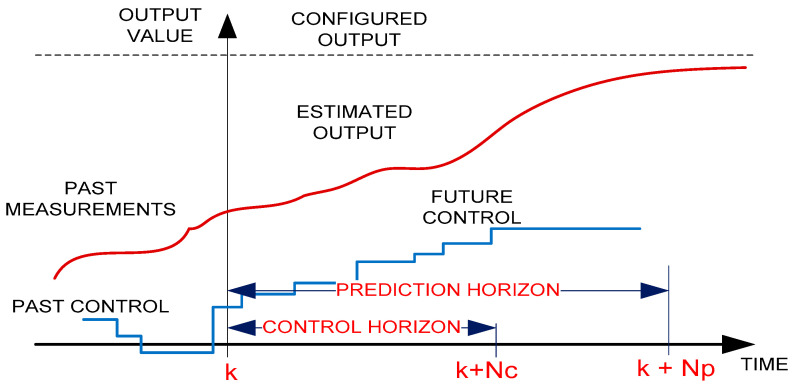
Prediction and Control Horizons.

Figure 11 shows these horizons.

In Figure 11, the mode of action of the prediction is presented as follows: for the sample k, at the time k + Np, the MPC controller predicts the output; at the next time k + 1, the MPC calculates the new output prediction value.

**Figure 12:**
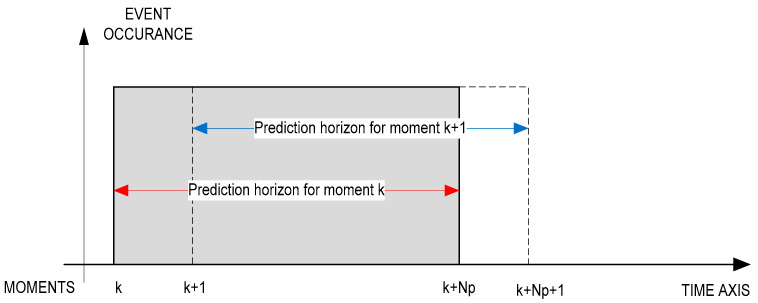
Moving the prediction horizon forward in time.

Figure 12 shows the evolution over time of the control, reference, and output quantities.

When adjusting the proportional physical processes, a value approximately equal to the duration of the index response of the process is recommended for the output prediction horizon. By choosing a significantly lower value of the prediction horizon, the control system becomes damped oscillator or ascending (unstable) oscillator.

Occasionally, to avoid the effect of large and sudden variations (step type) of the reference, a delay filter, of the first order, is used.

Next, we will consider the sampling period T = 1, when the length of the prediction horizon is equal to N.

Determining the optimal control on the output prediction horizon is performed by minimizing a square shape criterion (cost function) *J*(*k*):

(20) $$\begin{array}{cc} J\left(k\right)=\overset{N_{p}}{\underset{i=N_{w}}{\sum }}[\hat{y}(k & +i\left|k)\right.{ ]}^{T}Q\left[\hat{y}(k+i\lfloor k)-r(k+i\left|k)\right.\right]\\ & +\overset{N_{c}-1}{\underset{i=0}{\sum }}\left[\Delta u^{T}(k+i\left|k)\right.R\Delta u(k=i\left|k)\right.\right]\\ & +\overset{N_{p}}{\underset{i=N_{w}}{\sum }}{\left[u(k+i\left|k)-s(k+i\lfloor k)\right.\right]}^{T}N\left[u(k+i\left|k)-s(k+i\lfloor k)\right.\right] \end{array}$$

where:

- *k*—units of discretized time;
- *I*—the index the prediction horizon (no. of counts);
- *Np*—output prediction horizon;
- *Nw*—the start points of the prediction horizon;
- *Nc*—control horizon;
- *Q*—weight matrix output error;
- *R*—rate of change in control action weight matrix;
- *N*—the control action error weight matrix;
- $\hat{y}(k+i\left|k)\right.$—the sequence of future hypothetical values of the process output,
- estimated on the output prediction horizon based on the process model, previous output values and previous and future command values;
- $r(k+i\left|k)\right.$—the sequence of future values of the set size reference;
- $\Delta u(k=i\left|k)\right.$—the sequence of future values of the set size reference;
- $u(k+i\left|k)\right.$—is the sequence of free, applied incremental commands hypothetically on the horizon of free order;
- s(k+i⌊k)—the input setpoint.

The optimization method for the minimum and maximum predictions imposed as operating limits are used to determine the mode of operation in real conditions that are imposed by the process monitoring parameters. In this situation, the MPC control algorithm considers the minimum and maximum predictions imposed to the detriment of the determined output prediction. Under these conditions, the MPC controller adjusts the required minimum and maximum operating predictions such that these limits are not exceeded.

**Figure 13:**
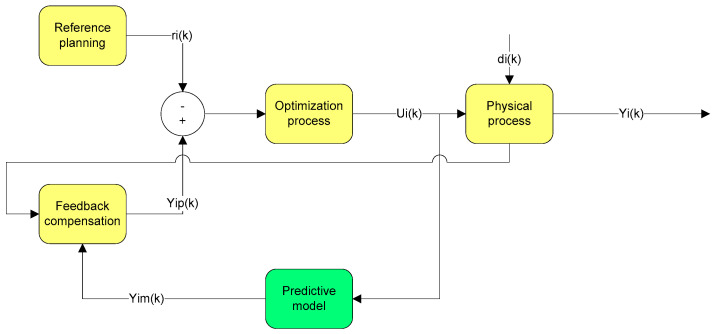
Flowchart of typical MPC.

Figure 13 shows the standard MPC architecture.

### 5.3. Proposed Framework

#### 5.3.1. Simulation and Experimental Verification of the Model

In this research, a new radial basic function model is presented, a predictive control model (RBFNN-MPC) for traffic flow and intrusions in data networks and for analyzing the proper functioning of servers using sensor systems (temperature and energy fluctuations). A novelty presented here is that the Hurst exponent is used to obtain local data traffic patterns on different network load modes.

Using the Hurst parameter, it is demonstrated that long-term dependence can be reduced by dividing the time series corresponding to each base station into series with a certain duration (e.g., one day). It can also be proven that daily traffic through a base station will not show the presence of long-term addiction. In a first stage, the Hurst exponent based on the wavelet, which is suitable for stationary time series, is determined. A strategy to select the mother wavelet functions is used based on their time-frequency location, because for communications traffic, location in time is the most important feature. Finally, for modeling the observations, an MPC algorithm is proposed based on a neural network with local radial base function (RBFNN), with self-organizing mechanism, and used for modeling local transfer functions to estimate the future behavior of the data network. This new algorithm was chosen because the data traffic management system in the network works online, and RBFNN follows the dynamics of the data traffic, while the use of the traditional MPC algorithm always uses a constant mathematical model.

**Figure 14:**
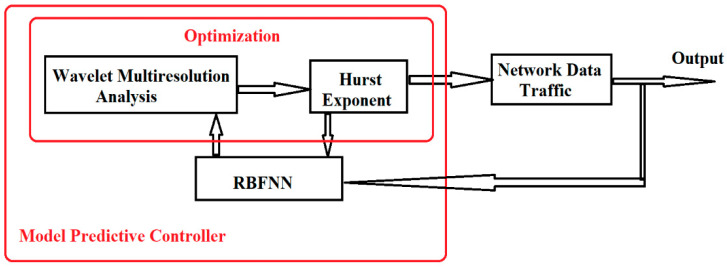
MPC based on RBFNN for each data network.

The architecture of the proposed algorithm (system) is presented in Figure 14.

For long-term network preventive maintenance purposes, or for improving its reliability and resilience, it is useful to develop an over-imposed AI application to monitor all failures causes and perform correlations between causes and effects, in order to keep record of major connections between failures and their effects in network services operation. Another added value will also be for the understanding of causes that produced a low availability/Apdex indexes over longer periods of time (at least a week, for example). This may be an important argument for reporting activities and future improvements of network resilience.

##### Algorithm’s Flow

**Figure 15:**
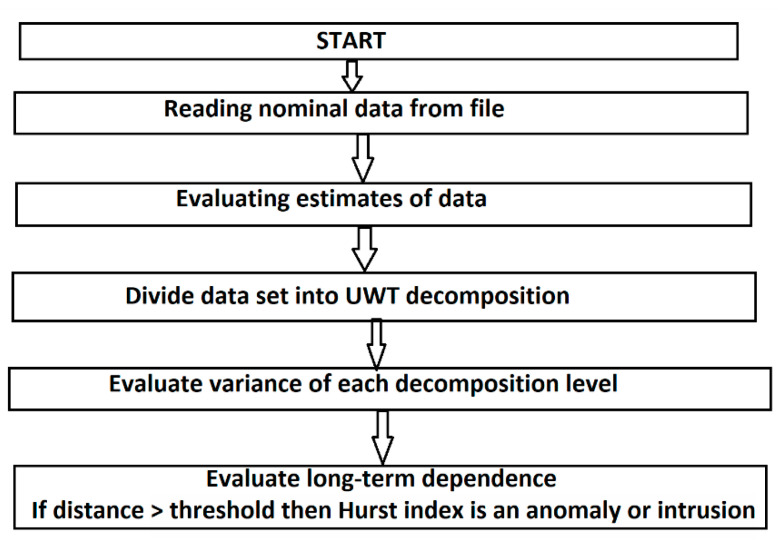
Algorithm’s flow for the proposed approach.

Figure 15 presents the calculation steps of the proposed algorithm. The process develops as follows:

- Reading of data.

In this step, the data files associated with the network data traffic are read from different monitored applications or hardware components.

- Evaluation of estimates.

Data set estimates are evaluated using MPC with RBF. Each data set is passed by estimation. RBFNN has three layers (input, hidden, and output) and four nodes corresponding to the four levels of UWT decomposition.

- Multi-resolution analysis.

The data is multi-resolution decomposed using UWT, and level 4 analysis

- Calculation of the Hurst index.

For each level of the UWT decomposition, the variation of the Hurst index is calculated.

- Long-Term Dependence assessment.

The absolute distance between the points is calculated to determine the short, medium, and long-term dependencies. This distance is plotted based on the analyzed period and the Hurst index.

The threshold is marked based on most occurrences of absolute distance differences. In most columns, this distance is less than 1 and 0.5. Based on the absolute distance, the decision criterion is taken. If the distance is less than or equal to the threshold value, the values of that window are classified as nominal and if the distance is greater than the threshold, we classify the values in those windows as anomaly or intrusion.

#### 5.3.2. Results Obtained for the Hurst Exponent and Multi-Resolution Wavelet Analysis

A high value of the Hurst exponent indicates desynchronization, thus, it must be ensured that the values obtained are higher for the signal corresponding to data networks with malfunctions and intrusions than for the signal corresponding to the normal operation of data traffic for C3 and CP3 networks and for the corresponding signal. The data network with malfunctions and intrusions is compared to the signal corresponding to a monitoring operation on the C4 and CP4 networks, in order to have a good discrimination of the transition elements, in which C3, C4, CP3, and CP4 are the indicators of the data networks for which we performed the tests using files obtained from the monitoring process.

**Figure 16:**
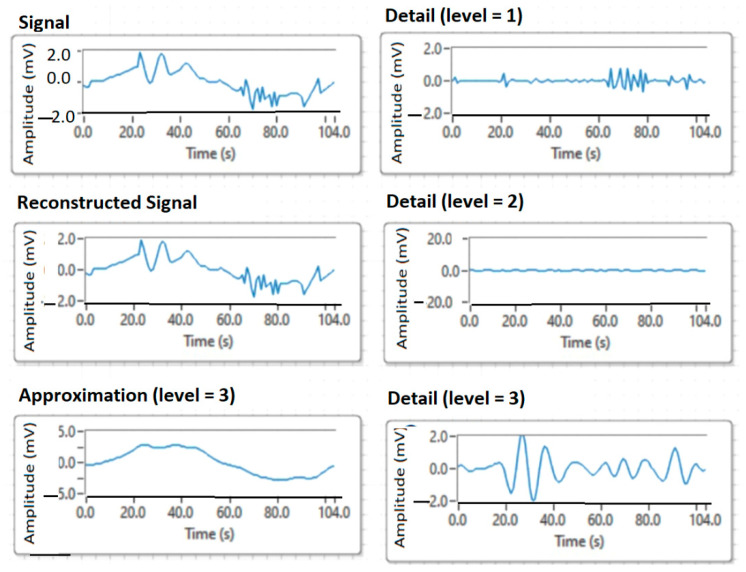
UWT analysis of the data network signal, using level 3 decomposition and ym6 function.

Figure 16 shows the level 3 decomposition using the wavelet symlet6 function. The figure shows the decomposition details highlighted on the three levels of analysis.

Such values are obtained with the Higuchi method, for both types of testing.

**Figure 17:**
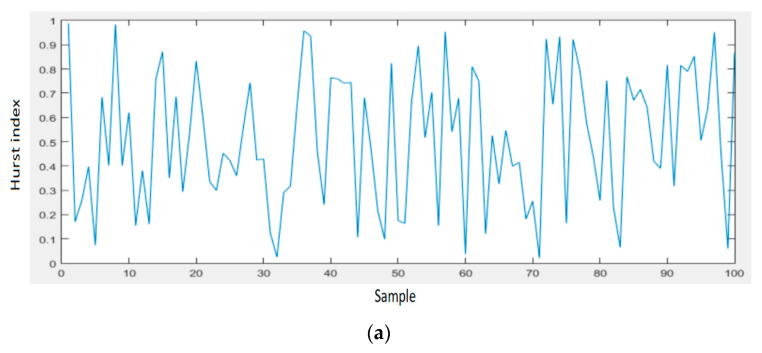

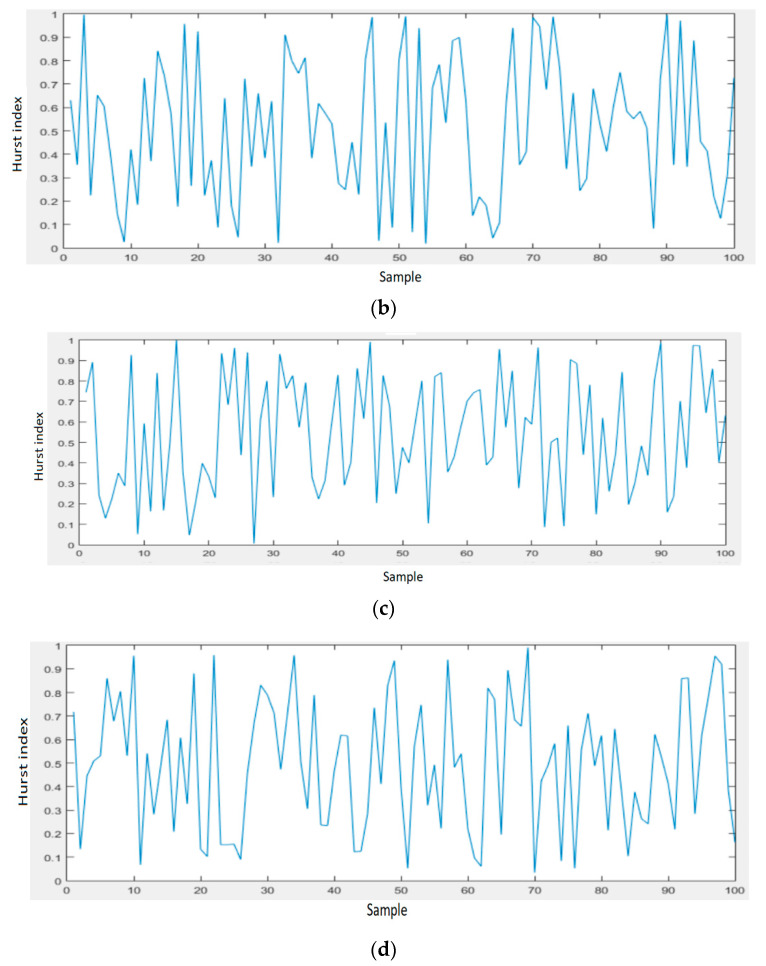
Representation and analysis of network traffic on the signal obtained from the decomposition on level 3, using the function sym6: (**a**) H = 0.39, (**b**) H = 0.46, (**c**) H = 0.29, and (**d**) H = 0.15.

Figure 17 shows the results of the Hurst exponent for signal samples related to a data network that highlights its proper functioning (the value of the Hurst exponent is small or close to zero), but also the occurrence of possible unauthorized faults or intrusions in the network when the value of the Hurst exponent is high, approaching the value of 0.5.

**Figure 18:**
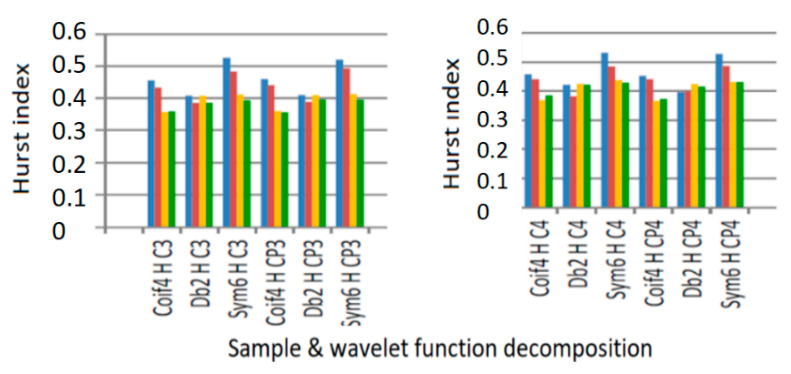
Graphical representation of the results obtained with all three wavelet functions tested for the signals corresponding to the operation of a network with malfunctions and intrusions compared to normal operations: (**a**) C3 and CP3 networks and (**b**) C4 and CP4 networks.

The results are represented graphically in Figure 18 for the wavelet methods and functions employed, separately for the normal mode of operation, and for the mode of operation with the occurrence of certain malfunctions and intrusions at the level of data networks.

The color code to be followed is the blue columns (corresponding to the malfunctioning network) which must be larger than the red (corresponding to the normally functioning network), and the yellow columns (corresponding to the malfunctioning and intrusion network) which must be larger than the green (corresponding to the normal operation of the network). In both figures, it is observed that all the blue columns, in all situations, are larger than the red ones.

Thus, we can conclude that the networks tested in the study for different families of wavelet functions (yellow columns) are larger mostly for the Hurst exponent determined with the Higuchi method, which proves to be the best method for calculating the Hurst exponent.

**Figure 19:**
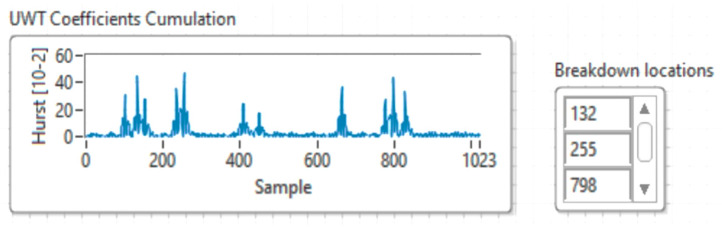
Traffic analysis for intrusion detection using UWT and the Hurst exponent.

Figure 19 shows the results of simulating an intrusion attack inside a data network based on UWT multiresolution analysis and the Hurst exponent.

In conclusion, extracting characteristics using the Hurst exponent is a method that should be considered when working with signals from data traffic during its use. However, it seems that the best method used to calculate the Hurst exponent is Higuchi. The use of the Hurst exponent calculated with the three methods of multi-resolution wavelet analysis, to highlight the characteristics of the recorded network traffic signals, is considered a significant contribution of this research. These methods have not been used thus far in determining the failures or intrusions in data traffic, for highlighting the proper functioning or malfunctioning of data networks.

To determine the time efficiency of the proposed method, the concept of long-range dependence (LRD) has been employed. Long-range dependence is also called long-term memory, being used in the analysis of spatial data and time series. LRD is based on the rate of decomposition of the statistical dependence between two points, over an increasing time interval (spatial distance between points). An event is considered to have long-range dependence if the dependence decomposes more slowly than exponential decomposition. LRD is used in financial predictions (econometrics), hydrology, and linguistics, but can be successfully applied for modeling traffic in data networks. For the LRD analysis, the Hurst parameter is calculated on the UWT multi-resolution decompositions applied to the signal corresponding to the data traffic in a network.

Given a stationary LRD sequence, the partial sum, if viewed as a process indexed by the number of terms after proper scaling, is a process similar to asymptotic stationary increases.

**Figure 20:**
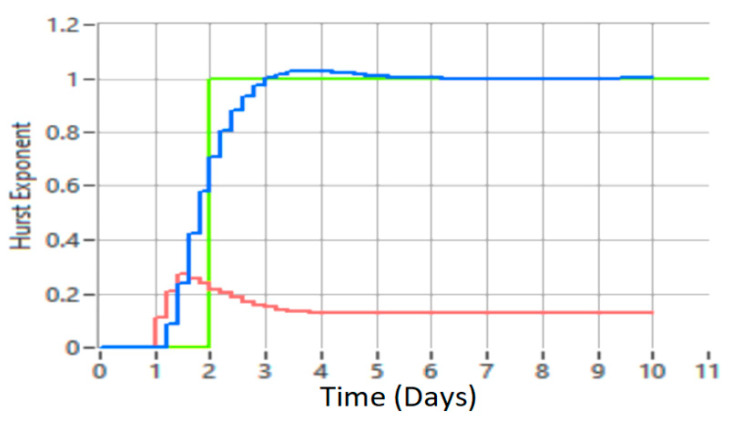
Comparison between the proposed MPC method with RBFNN and the classic MPC method (reference—green color, proposed method—blue color, and classic MPC method—red color).

**Figure 21:**
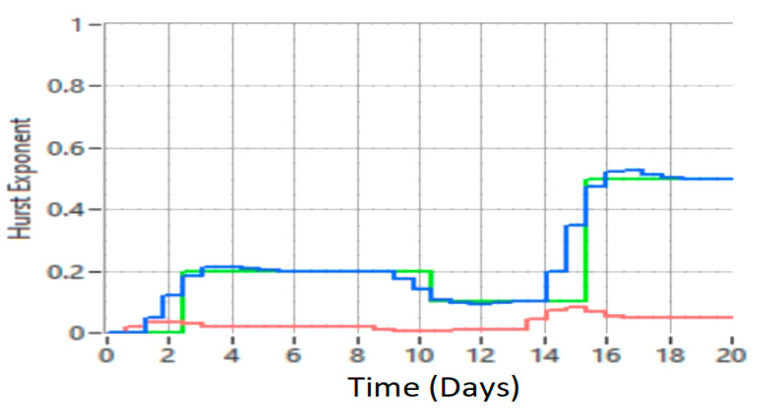
Detection of failures and intrusions in the data network traffic obtained with the proposed method (blue line) compared to the classic MPC method (red line). The green line represents the reference.

**Figure 22:**
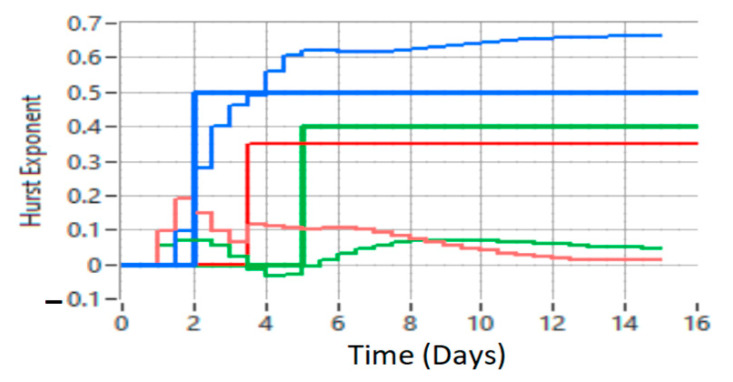
Analysis of the operation of three data networks: network 1 (blue line), network 2 (green line), and network 3 (red line).

The Hurst parameter can be called the dependency index or the long-range dependency index. This parameter quantifies the tendency of a time series associated with a process to either regress steeply to the mean or to group in a predictive direction. Thus, the value of the Hurst parameter (Hurst index) in the range 0.5–1 indicates a time series with long-term positive autocorrelation, possibly a high value to be followed by another high value, thus indicating a desynchronization of data traffic. If the Hurst index has the value 0.5 then the series is completely uncorrelated, thus establishing the comparison value. A value in the range of 0–0.5 indicates a time series with long-term switching between the highest and lowest values between adjacent pairs, which means that a single high value is likely to be followed by a low value and that the value after that it will tend to be high, with this tendency to switch between high values and low values that last a long time in the future. H values in the range of 0–0.5 are interpreted as a series of average (anti-persistent) recovery. The closer the value is to 0, the stronger the average reversal process. In practice, the value of H in the range of 0–0.5 corresponds to a normal operation of traffic in data networks. The results of applying the LRD concept with the proposed method are presented in Figure 20, Figure 21 and Figure 22.

Next, achieving the proposed MPC based on neural network is compared with radial function with optimization, performed with wavelet functions and the Hurst exponent, with conventional MPC. Following the simulations and tests performed on data traffic records and the proposed method employing RBF neural networks, better results were obtained in reducing uncertainty than by the traditional method. Figure 20 shows the control of data network traffic by the proposed method (MPC based on RBFNN) and the classical MPC method without uncertainties.

For a better presentation, Figure 21 shows the detection of a defect or intrusion (blue graph), compared to the interpretation of data traffic with the classic MPC method (red line).

Comparing Figure 20 and Figure 21, the results indicate that the proposed method is superior to the classical MPC method.

The results shown in Figure 21 suggest that for the classical MPC method, the range of the Hurst exponent is 0–0.15, while in the case of the proposed method the range is 0–0.5, which demonstrates that the classical MPC method cannot detect faults or intrusions in the comparative network. With the proposed method it is possible to highlight defects and intrusions in the network and return it to a normal operation.

From Figure 20 and Figure 21, it can be concluded that the higher the data network traffic load, the better the results obtained with the proposed MPC method with the RBF neural network compared to the classical MPC method.

Figure 22 shows the analysis of samples recorded for three data networks using the proposed method.

The interpretation of Figure 22 shows the malfunction of network 1 (blue graph), with malfunctions or possible unauthorized intrusions into the network, the normal operation of network 2 (green graph), and the partially normal operation of network 3 (red graph). By interpreting these three graphs we can conclude that the use of intelligent computational algorithms together with a predictive control model that employs multiresolution and Hurst exponent decompositions for time series analysis is a useful system control solution. Data network traffic and its ability to load workstations with tasks represents a nonlinear system with uncertain parameters. As it is shown in the results obtained from the simulations, a real monitoring of a data network traffic cannot be approximated with a fixed model and with fixed parameters.

Instead, by combining the classical MPC method with intelligent analysis algorithms, as in the proposed method, network changes (failures, anomalies, and intrusions) can be detected in real time. Neural networks with radial function, if they are driven with correct data, can approximate a function and can estimate future moments according to the horizon prediction and control model, and this aspect is useful in controlling systems.

## 6. Discussion and Conclusions

Based on a case study of practical management of applications monitoring in highly responsive networks, in this work a solution for improving communications network’s fault maintenance and resilience has been proposed. With the actual growth in services demand and data communications, it is considered that maintenance process importance will increase significantly. Maintaining high levels of quality for communications will also become a critical task. The case study was performed for specific applications monitoring, employing a Davis AI engine with a combination of artificial intelligence and human operations. The case showed that, when using classical AI-based instruments for monitoring applications, there still may occur situations when the recovery process takes longer than usual, due to chain events that cannot be monitored by causality. Therefore, in the second part of this paperwork an over-imposed solution is proposed for tracking and storing knowledge about such types of events, a solution also based on machine-learning, meant to further improve the performances of fault management and recovery operations.

For defining a methodology able to detect abnormal functioning of different components of a communications network, a combination of computational intelligence with model predictive control was used to analyze network data traffic. The proposed solution uses online traffic modeling employing a neural network with a radial base function. In the first step, several local transfer functions were created for the network traffic using wavelet multiresolution analysis together with the Hurst exponent calculation; then, an RBF neural network was used to approximate these models. RBFNN can estimate future moments for the predictive method of the model and can be used to accurately control the proper functioning of the data network. It is also able to highlight the occurrence of network defects, or unauthorized intrusions. The simulation results reveal that the proposed method using the predictive control of the neural network-based model works better than the classical predictive control, especially when the uncertainty is high. All numerical values of parameters and their mathematical relationships are based on the real-time operation of a data network, using real signal samples. As a result, the method proposed in this research prove to have the ability to implement hardware and software for quasi-real-time data traffic sensing and monitoring.

The following are contributions in this research:

- a specific case study was performed for a mobile communications network failure, considering conditions, environment, and comparative recovery time;
- an analysis of existing AI efficiency in discovering and analyzing network applications failures, with comparative availability indexes, level of service, and quality factors;
- a proposal for a new approach to determine network applications and hardware failures, based on extended AI, and associated machine learning techniques;
- a comparative analysis of the fourth order decomposition of the wavelet coefficients, determining for each decomposition the estimation of the Hurst parameter based on the wavelet, a useful method for the analysis of the time series;
- a strategy for selecting mother wavelet functions, based on their location and frequency has been developed;
- a demonstration through simulations that, in the situation of communications traffic, the location in time is important to choose the mother wavelet functions;
- demonstrating that the best results are obtained with the help of the sym8 wavelet wave (eighth order symlet); this is due to the invariance of its wavelet functions against translations;
- demonstrating, through simulations, that the multi-resolution wavelet decomposition is able to predict data traffic for data radio communications (Wi-Fi, WiMAX, GSM). This is due to its invariance in translations;
- based on the Hurst exponent, it has been shown that long-term dependence is reduced by dividing the time series corresponding to each base station into one-day series;
- the increase in value of the Hurst exponent is the result of the appearance of certain unauthorized network anomalies or intrusions that may occur during certain periods;
- Using the concept of long-range dependence based on the Hurst exponent and UWT decomposition, with reference to time (or long-term reference).

The algorithm proposed in this paper was tested off-line on files with data collected from four data networks, and not tested in real conditions. However, real-time intrusions into data networks were simulated, and the analysis of these traffic files using the proposed algorithm highlighted the intrusions and the predictability of their occurrence.

Using the proposed method, we estimate that it is possible to efficiently locate signals related to time-frequency failures and intrusions, also when if the signal is not predominant and persistent. For a more efficient localization of failures and intrusions we will continue the development of the proposed algorithms by creating a detection methodology for imposed structures, with applicability in the time-frequency domain. It is also envisaged to test the proposed algorithm in real time, with the support of a cybersecurity company.

## Figures and Tables

**Table 1:** Applications of the wavelet transform for the analysis of traffic in the communications networks.

Crt.no.	Monitored Application	Wavelet Transform	Ref.
1	Network traffic anomaly detection	DWT	[1]
2	Packet length detection	DWT	[2]
3	Network intrusion detection	DWT	[3]
4	Cellular metric smoothing	DoM, Wavelet	[4]
5	Degradation identification	FFT	[5]
6	Traffic identification	Wavelet	[6]
7	Level prediction	FFT	[7]

**Table 2:** A summary of the methods used for fault/anomaly detection.

Application	Techniques	Short description	Ref.
Manual thresholding	Entropy State Machine	Identification degradation interval using manually thresholding	[13]
Statistical thresholding	Average Probability	Using statistical thresholding or average of the samples	[17,18]
Patterns comparation	Clustering Correlation	Analysis of time-series with normal/faults patterns	[8]
Predictor	ARMA ARIMA LSTM	Using predicted metrics and observed degradation score	[19,20,21]
ML	SVM, ANN Clustering	Training data with normal/fault patterns and classification using ANN	[8,12,18]

**Table 3:** Analysis of availability as shown by intelligent agent Davis AI.

Crt.no.	Monitored Application	Availability Index (%)	Total Minutes of Application’s Unavailability out of 172,800 (min)
1	A1	99.97	49
2	A2	99.82	313
3	A3	99.15	765
4	A4	99.45	558
5	A5	99.87	228
6	A6	99.91	135
7	A7	99.73	393
8	A8	99.25	731
9	A9	99.42	585
10	A10	99.77	367

## Data Availability

The data used in this study were obtained in the tests performed in the Artificial Intelligence laboratory within the Faculty of Transport, Polytechnic University of Bucharest, Romania.
